# Association between serum amylase levels and CD4 cell counts in newly diagnosed people living with HIV: A case-control study

**DOI:** 10.1097/MD.0000000000032638

**Published:** 2023-01-13

**Authors:** Yong Jin, Tianmeng Yang, Ting Xia, Zhihong Shen, Tingting Ma

**Affiliations:** a Department of Internal Medicine, Ningbo Yinzhou No.2 Hospital, Ningbo, People’s Republic of China.

**Keywords:** CD4 cell counts, HIV, newly diagnosed, serum amylase

## Abstract

Serum amylase is a direct reflection of pancreatic injury. Several clinical studies have indicated that antiretroviral therapy may be the main cause of increased serum amylase in people living with human immunodeficiency virus (PLWH). However, other probable causes including direct human immunodeficiency virus infection, opportunistic infections and neoplasms, alcohol abuse, and use of illicit drugs, which can also affect pancreatic amylase levels were not considered in these studies. In our study, we collected clinical data from newly diagnosed PLWH who had not received antiretroviral therapy, and examined the association between serum amylase levels and CD4 cell counts. Between November 2018 and September 2021, a total of 344 newly diagnosed PLWH and 344 healthy controls were recruited at Ningbo Yinzhou No 2 Hospital. Serum amylase levels, CD4 cell counts and other clinical features were measured. Relationships between serum amylase levels and clinical parameters were evaluated using correlation analysis. Multiple linear regression analyses were performed to identify the independent risk factors. Newly diagnosed PLWH had lower CD4 cell counts and higher serum amylase levels than healthy controls (*P* < .05). Serum amylase levels were negatively correlated with CD4 cell counts (*r* = −0.506, *P* < .001). In multiple linear regression analyses, CD4 cell counts (β = −0.327, 95% confidence interval = −0.051–−0.022, *P* < .001) were independently associated with serum amylase levels. CD4 cell counts were independently associated with serum amylase levels in newly diagnosed PLWH. Thus, close monitoring of serum amylase may be significant in preventing opportunistic infections of PLWH, since low CD4 cell counts are associated with an increased risk of opportunistic infections.

## 1. Introduction

Human immunodeficiency virus (HIV) infection and acquired immunodeficiency syndrome (AIDS) are global health issues. According to the Joint United Nations Programme on HIV/AIDS, 38 million people were living with HIV by 2020 (https://www.unaids.org/en/resources/fact-sheet). Due to the widespread availability of antiviral therapy, the number of people living with HIV (PLWH) is expected to continue to increase.^[1,2]^ Altered serum amylase levels are a common phenomenon in PLWH.^[3]^ Amylase is mainly secreted by the pancreas, although a small amount is also secreted by the salivary glands, proximal duodenum, lung, uterus, mammary glands during lactation, and other organs. The main function of amylase is to decompose polysaccharides, such as starch and glycogen. Pancreatic damage is first considered when the serum amylase concentration increases. Pancreatic tissue damage in the initial phase is a reversible process, and milder inflammatory damage can be recovered through autocompensation, thus manifesting only as increased serum amylase. Acute pancreatitis develops only when the injury reaches a certain severity and causes irreversible necrotic damage to the pancreatic parenchyma, accompanied by a sharp rise in serum amylase (concentration is >3 times of normal level). In this regard, pancreatic involvement in PLWH and the higher probability of diabetes in these people have been widely studied and reported.^[4]^ And the incidence of pancreatitis in PLWH is significantly higher than that in healthy people.^[5]^ It suggests that the actual incidence of pancreatic exocrine impairment may be underestimated. In addition, direct HIV infection, opportunistic infections, neoplasms, alcohol abuse, use of illicit drugs, aging, history of pancreatic disease and various treatments for PLWH including antiretroviral therapy (ART), other antiviral treatments and anti-tuberculosis drugs may contribute to subclinical and clinical pancreatic damage (including hyperamylasemia and acute pancreatitis).^[6]^ A single factor in the evaluation of pancreatic exocrine impairment in PLWH faces difficulties.

The introduction of ART has improved the life expectancy of PLWH significantly. ART alters the natural outcome of AIDS, making it a chronic, treatable disease. At the same time, drug-related complications cannot be ignored. Initial ART includes 2 nucleoside reverse transcriptase inhibitors (NRTIs) and a third agent (e.g., non-NRTIs, protease inhibitors or integrase inhibitors). Studies have demonstrated that single or dual combinations of NRTIs may increase the incidence of acute pancreatitis and hyperamylasemia.^[7,8]^ The mitochondrial toxicity of NRTIs to pancreatic cells is one of the possible mechanisms.^[9]^ The role of protease inhibitors in acute pancreatitis is controversial, and it may cause the incidence of acute pancreatitis by increasing serum triglyceride levels.^[8,10]^ Because ART is a long-term therapeutic strategy for PLWH, the clinical significance of laboratory amylase abnormalities warrants reassessment.

During HIV infection, HIV damages the host immune system by inducing CD4 cell apoptosis, eventually leading to immunodeficiency and opportunistic infections.^[11,12]^ CD4 cell counts in HIV patients are closely related to disease progression.^[11–13]^ However, in newly diagnosed PLWH, CD4 cell counts are often lower than normal at the time of diagnosis, and the degree of decline in CD4 cell counts is often associated with a delay in diagnosis.^[14]^ Delayed diagnosis of HIV means an increased risk of opportunistic infections and a corresponding reduction in survival time. Therefore, CD4 cell counts is considered to be an important observation index in clinical practice and many HIV-related studies.

Although many earlier studies have highlighted the effect of ART or other drugs on serum amylase levels, few studies have examined the effects of disease status per se on serum amylase levels in PLWH. In addition, changes in serum amylase levels in PLWH in the Chinese population have not yet been reported. Thus, in the present study, we performed a case-control study to evaluate the relationship between serum amylase levels and CD4 cell counts in newly diagnosed PLWH and to further understand the pathophysiological mechanism of HIV.

## 2. Subjects and methods

### 2.1. Study subjects

The case-control study consecutively enrolled newly diagnosed PLWH aged 18 to 75 years from Ningbo Yinzhou No.2 Hospital, between November 2018 and September 2021. The inclusion criteria were as follows: positive HIV serology results and newly diagnosed PLWH, not previously treated with ART. The exclusion criteria were as follows: patients with a history of taking medication that may affect serum amylase levels or CD4 cell counts; history of pancreatitis or pancreatic tumor or pancreatic surgery; history of alcohol abuse; patients with a history of neoplasm; and overt infection. A total of 344 eligible patients were enrolled (304 men and 40 women, with a mean age of 38.3 ± 14.9 years) and were assigned to the case group. The control group consisted of healthy people undergoing health examinations. Each newly diagnosed PLWH was matched with 1 healthy control of the same age and sex. Written informed consent was acquired from each participant, and the study was endorsed by the Ethics Committee of the Ningbo Yinzhou No.2 Hospital (no. 2019-R057). The purposes and procedures of the study were explained to the participants prior to the questionnaire and blood data collection.

### 2.2. Clinical and laboratory examination

Patient information including age, sex, concomitant illnesses and medical history was obtained from the self-reported questionnaire. Body weight was measured with a weighing machine (to the nearest 0.1 kg), while the subjects were barefoot and wearing light indoor clothing. Blood samples were collected after an overnight fasting period. All patients were subjected to the following laboratory tests: CD4 cell counts, CD8 T cell counts, viral load, white blood cell (WBC) counts, and levels of lymphocytes (LY), hemoglobin (HGB), platelets (PLT), alanine aminotransferase, aspartate aminotransferase (AST), total cholesterol, and triglyceride. All biochemical parameters were detected by standard automated laboratory methods using commercially available kits in accordance with the manufacturer’s protocols.

### 2.3. Statistical analysis

Statistical analyses were performed using SPSS software (SPSS Inc., Chicago, IL) in version 24.0 for Mac. Before proceeding with the statistical analysis, all the parameters were tested for a normal distribution using the Kolmogorov–Smirnov test. The above parameters contain non-normally distributed variables and variables were expressed as the median (inter-quartile range). The Mann–Whitney test was used to compare variables between the 2 groups. Categorical variables were compared using the *χ*^2^ test. The relationships between serum amylase levels and clinical parameters were evaluated using Spearman correlation analysis. Multiple linear regression analysis was used to assess the independent correlation between CD4 cell counts and serum amylase levels. Two-tailed *P* < .05 was considered statistically significant.

## 3. Results

### 3.1. Anthropometric and biochemical characteristics of the study population

A total of 344 newly diagnosed PLWH and 344 matched controls who met the inclusion and exclusion criteria were included in this study. Anthropometric indices and clinical characteristics, as well as laboratory measurements of the study population are shown in Table 1. There were no differences in sex, age, weight, CD8^+^T cell counts, and levels of WBC, HGB, PLT, alanine aminotransferase, and AST between the 2 groups (*P* > .05). However, compared to the controls, PLWH had lower CD4 cell counts (*P* < .05). In addition, PLWH had higher total cholesterol and serum amylase levels and lower LY levels compared with the control group (*P* < .05).

**Table 1:** Anthropometric and biochemical characteristics of PLWH and controls.

Characteristic	Case group (n = 344)	Control group (n = 344)	*P* value
Male/female	304/40	304/40	>.99
Age (yr)	35 (25,49)	38 (30,44)	.327
Weight (kg)	63 (58,68)	62 (55,69)	.362
CD4 cell counts (cells/µL)	237 (158,319)	1008 (771,1276)	<.001
CD8^+^T cell counts (cells/µL)	660 (491,916)	687 (466,925)	.681
Log viral load (copies/mL)	5 (4,5)	–	–
WBC (×10^9^)	5 (4,6)	5 (4,6)	.343
LY (×10^9^)	2 (1,2)	3 (2,5)	<.001
HGB (×10^9^)	146 (135,156)	146 (128,163)	.357
PLT (×10^9^)	210 (173,245)	213 (187,241)	.262
ALT (U/L)	20 (14,30)	21 (11,32)	.105
AST (U/L)	21 (17,26)	21 (9,33)	.141
TC (mmol/L)	4 (3,5)	3 (2,4)	<.001
TG (mmol/L)	1 (1,2)	1 (1,2)	.315
Serum amylase (U/L)	74 (69,79)	48 (36,59)	<.001

ALT = alanine aminotransferase, AST = aspartate aminotransferase, HGB = hemoglobin, HIV = human immunodeficiency virus, LY = lymphocytes, PLT = platelets, PLWH = people living with HIV, TC = total cholesterol, TG = triglyceride, WBC = white blood cell.

### 3.2. Correlation analysis of the anthropometric data, laboratory measurements and serum amylase levels

Spearman correlation analysis was performed between serum amylase levels and clinical indicators in newly diagnosed PLWH (Table 2). The results showed serum amylase levels was positively associated with age, log viral load and AST levels and negatively associated with CD4 cell counts, CD8 cell counts, WBC levels, LY levels, HGB levels, and PLT levels (*P* < .05), but was not associated with other variables (*P* > .05).

**Table 2:** Association between serum amylase levels and the anthropometric and laboratory characteristics in newly diagnosed PLWH.

Variable	*r*	*P* value
Age (yr)	0.182	.001
Weight (kg)	-0.093	.087
CD4 cell counts (cells/mL)	-0.506	<.001
CD8 cell counts (cells/mL)	-0.156	.004
Log viral load (units/mL)	0.141	.009
WBC (×10^9^)	-0.207	<.001
LY (×10^9^)	-0.203	<.001
HGB (×10^9^)	-0.183	.001
PLT (×10^9^)	-0.144	.007
ALT (U/L)	0.069	.201
AST (U/L)	0.197	<.001
TC (mmol/L)	-0.088	.103
TG (mmol/L)	0.029	.595

ALT = alanine aminotransferase, AST = aspartate aminotransferase, HGB = hemoglobin, HIV = human immunodeficiency virus, LY = lymphocytes, PLT = platelets, PLWH = people living with HIV, TC = total cholesterol, TG = triglyceride, WBC = white blood cell.

### 3.3. Multivariate linear regression analysis regarding the association of CD4 cell counts with serum amylase levels

CD4 cell counts and AST were examined by multivariate linear regression analysis (Table 3). When the variables were unadjusted (model 1), CD4 cell counts was found to be positively correlated with serum amylase (*β* = −0.324, 95% confidence interval [CI] = −0.047–−0.025, *P* < .001). The correlation was still significant (*β* = −0.314, 95% CI = −0.046–−0.023, *P* < .001) after further adjustment for age (model 2). When age, CD8 cell counts, log viral load, WBC, LY, HGB, PLT, and AST were included in the linear regression model (model 3), the correlation between CD4 cell counts and serum amylase was also significant (*β* = −0.327, 95% CI = −0.051–−0.022, *P* < .001).

**Table 3:** Multiple linear regression analyses of variables associated with serum amylase levels in newly diagnosed PLWH.

	Model 1			Model 2			Model 3		
Variable	*β*	95% CI	*P* value	*β*	95% CI	*P* value	*β*	95% CI	*P* value
CD4 cell counts	−0.324	−0.047 to −0.025	<.001	−0.314	−0.046 to −0.023	<.001	−0.327	−0.051 to −0.022	<.001
Age				0.038	−0.069–0.150	.470	0.065	−0.002–0.007	.284
CD8 cell counts							0.075	−0.482–3.113	.151
Log viral load							−0.030	−1.572–0.948	.627
WBC							0.001	−0.197–0.200	.989
LY							0.068	−0.037–0.152	.233
HGB							0.052	−0.016–0.045	.345
PLT							0.099	−0.005–0.159	.065
AST							0.058	−0.053–0.175	.295
Adjusted *R*^2^	0.103			0.101			0.106		

Model 1 was unadjusted. Model 2 was adjusted for age. Model 3 was adjusted for age, CD8 cell counts, log viral load, WBC, LY, HGB, PLT and AST.

AST = aspartate aminotransferase, CI = confidence interval, HGB = hemoglobin, HIV = human immunodeficiency virus, LY = lymphocytes, PLT = platelets, PLWH = people living with HIV, WBC = white blood cell.

## 4. Discussion

Our study examined the association between CD4 cell counts and serum amylase levels in newly diagnosed, Chinese PLWH using a case-control study design. To the best of our knowledge, this is the first study to specifically address the relationship between CD4 cell counts and serum amylase levels in newly diagnosed PLWH. We found that newly diagnosed PLWH had lower CD4 cell counts and higher serum amylase levels compared to healthy controls. Furthermore, correlation analyses also demonstrated that CD4 cell counts were significantly and negatively correlated with serum amylase levels. After adjustment of potential confounding factors, multivariable linear regression analyses showed that CD4 cell counts were independently and negatively associated with serum amylase levels.

Although several studies have previously reported the association between CD4 cell counts and serum amylase levels, the results were somewhat controversial and limited. For example, Argiris et al^[15]^ reported that PLWH with higher serum amylase levels had lower CD4 cell counts. However, there was no independent association of CD4 cell counts with serum amylase levels in the logistic regression model. While Chehter et al^[16]^ demonstrated that serum amylase levels did not differ significantly among the groups separated according to CD4 cell counts in PLWH. Riedel et al^[8]^ showed that CD4 cell counts of <50 cells/µL were a risk factor for increased serum amylase levels by multivariate regression analyses. The study also indicated that there was an association between the female sex and increased serum amylase levels. Similarly, Dragovic et al^[17]^ reported that female sex and CD4 cell counts of <200 cells/µL were risk factors for acute pancreatitis in addition to ART. The differences in the findings of the above studies may be due to differences in sex, age, and inclusion and exclusion criteria. Compared with previous studies, our study was unique for several reasons. First, our research was conducted on Chinese patients. Secondly, we included newly diagnosed PLWH in our study to eliminate the influence of ART or other medications that may affect serum amylase levels and CD4 cell counts. In addition, PLWH were grouped according to their serum amylase levels to further examine the clinical characteristics of the different groups. Finally, we conducted stepwise multivariate analyses to further confirm the association of CD4 cell counts with serum amylase levels and to eliminate the influence of other confounding factors.

At present, the mechanism by which CD4 cells affect serum amylase levels remains unclear and the direct pancreatic damage caused by the virus may be one of the important reasons. It is well known that the pancreas itself has a constant microbiota physiologically.^[18]^ Microorganisms in the pancreas were the key to viral pancreatitis. Studies have shown that a wide spectrum of viruses, such as HIV, severe acute respiratory syndrome coronavirus 2, hepatotropic virus, Cytomegalovirus, Ebola virus, Coxsackie virus, Paramyxovirus, Herpes Simplex virus and Varicella Zoster virus, as well as tuberculosis, *Mycobacterium* and Toxoplasmosis, can infect pancreatic cells and present clinical manifestations ranging from mild to severe.^[19–26]^ Microorganisms in the pancreas of PLWH have a unique composition compared to the pancreas of a healthy population. Early studies of necropsy AIDS patients have demonstrated the presence of microorganisms. A necropsy report by Chehter et al^[27]^ demonstrated that mycobacteriosis (22%), toxoplasmosis (13%), cytomegalovirus (9%), *Pneumocystis carinii* (9%), and HIV (22%) were observed in the cytoplasm of macrophages in the pancreas from AIDS patients. And the CD4 cell count of the above-mentioned patients was 102 ± 155 cells/µL, which was significantly lower than normal. When immune dysfunction, pancreatic microorganisms from colonization to infection progress.^[28,29]^ In fact, in a recent systematic review of viral-attributed acute pancreatitis, a significant number of patients (28.0%) were immunocompromised and this proportion accounted for a significantly higher percentage of deaths (71.4%).^[30]^ In our study, newly diagnosed PLWH were used as the research subjects so that the effects of HIV drugs on exocrine pancreatic injury could be excluded. Combined with alterations in CD4 cell counts and previous literature reports, we hypothesized that a decrease in CD4 cell counts leads to opportunistic infections of microorganisms colonized in the pancreas,^[31]^ which damage the pancreas or other organs and cause asymptomatic increases in serum amylase. Since we excluded patients with the overt infection before the start of the study, we tend to think that microorganisms in the pancreas are latent infection. However, further research is still needed to clarify our initial hypothesis due to some other limiting factors in this study.

Some limitations in our research should be recognized. First, the sample size of our study was relatively small and the results were based on patients from a single institution. Since the newly diagnosed PLWH with acute pancreatitis is rare and it only appears in a case report.^[22]^ Our study subjects did not include patients with acute pancreatitis. Second, elevated serum amylase levels are usually due to pancreatic damage, and other causes including gastrointestinal diseases, macroamylasemia, acidemia, renal failure, and parotid gland disease cannot be ignored. Thus, it is necessary to evaluate pancreatic damage with serum lipase levels or pathological biopsies in subsequent studies. In addition, the current study did not demonstrate a causal relationship between CD4 cell counts and serum amylase levels or potential pancreatic damage. Thus, larger prospective studies are warranted to provide more definitive evidence.

## 5. Conclusion

Using newly diagnosed PLWH to exclude the interference of ART and other anti-HIV drugs on serum amylase levels, we designed a cross-sectional study to verify the correlation between serum amylase levels and CD4 cell counts. Furthermore, we speculated that opportunistic infections caused by low CD4 cell counts may be the main cause of pancreatic damage in PLWH. Thus, the possibility of pancreatic involvement should constantly be considered in PLWH related infections. Close monitoring of serum amylase levels may be important for the detection of opportunistic infections in PLWH.

## Acknowledgment

We profoundly thank Dr Shuqin Chen for her contribution to this research.

## Author contributions

**Conceptualization**: Yong Jin, Tingting Ma.

**Data curation:** Tianmeng Yang, Ting Xia.

**Formal analysis:** Ting Xia.

**Funding acquisition:** Zhihong Shen, Tingting Ma.

**Investigation:** Ting Xia.

**Visualization:** Zhihong Shen.

**Writing – original draft:** Yong Jin.

**Writing – review & editing:** Yong Jin, Tianmeng Yang.
